# Bioinformatics and the Metaverse: Are We Ready?

**DOI:** 10.3389/fbinf.2022.863676

**Published:** 2022-05-12

**Authors:** Stephen Taylor, Shamit Soneji

**Affiliations:** ^1^ Analysis, Visualization and Informatics Group, MRC Weatherall Institute of Computational Biology, MRC Weatherall Institute of Molecular Medicine, Oxford, United Kingdom; ^2^ Division of Molecular Hematology, Department of Laboratory Medicine, Faculty of Medicine, BMC, Lund University, Lund, Sweden; ^3^ Lund Stem Cell Center, Faculty of Medicine, BMC, Lund University, Lund, Sweden

**Keywords:** metaverse, virtual reality, augmented reality, mixed reality, visualization, immersive, bioinformatcs

## Abstract

COVID-19 forced humanity to think about new ways of working globally without physically being present with other people, and eXtended Reality (XR) systems (defined as Virtual Reality, Augmented Reality and Mixed Reality) offer a potentially elegant solution. Previously seen as mainly for gaming, commercial and research institutions are investigating XR solutions to solve real world problems from training, simulation, mental health, data analysis, and studying disease progression. More recently large corporations such as Microsoft and Meta have announced they are developing the Metaverse as a new paradigm to interact with the digital world. This article will look at how visualization can leverage the Metaverse in bioinformatics research, the pros and cons of this technology, and what the future may hold.

## Introduction

The “Metaverse” is based on the 1992 novel “Snow Crash” by Neil Stephensen. The novel depicts a hierarchical corporate-led dystopia, but many institutions and companies see this as a “new” internet: an open and inclusive virtual environment that offers new opportunities to understand data using visualization. For example, using a Head Mounted Display (HMD) geographically widespread users (represented by avatars) can interact as if they were in the same room and virtual objects can be manipulated using controllers or hand gestures in 3D space. The types of HMDs govern the eXtended Reality (XR) experience. These are summarized in Table 1.

**TABLE 1:** Summary of types of XR and their advantages/disadvantages (adapted from https://www.forbes.com/sites/quora/2018/02/02/the-difference-between-virtual-reality-augmented-reality-and-mixed-reality/?sh=6880b402d07c).

	Virtual reality (VR)	Augmented reality (AR)	Mixed reality (MR)
What is it	Fully artificial environment	Virtual objects overlaid on a real world environment	Virtual environment combined with real world 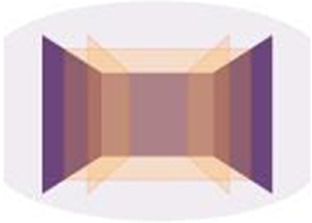
	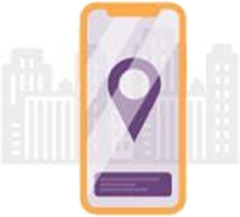	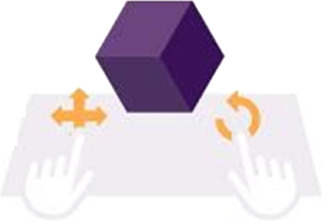	
Experience	Full immersion in a virtual environment 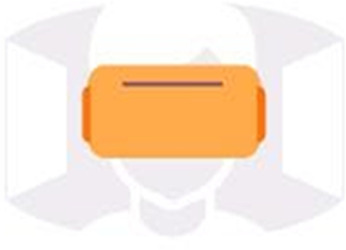	The real world enhanced with digital objects	Interact with both the real world and the virtual environment 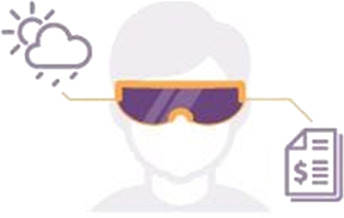
		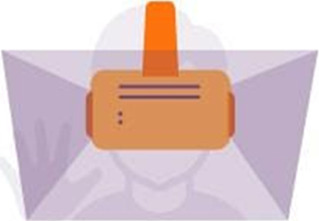	
Models	HTC Vive, Valve Index, Meta Quest 2, Meta Rift, PiMax, HP Reverb, Varjo, Pico Neo 2	Mobile phones, Snapchat glasses	(a) Projection based:
			Hololens 2, NReal (b) Camera Based
			Varjo, Quest 2
Pros	Most mature technology, least expensive	User is less isolated from environment; useful for training by overlaying instructions on the display; user has existing hardware	User can see the environment and can engage with other items e.g., mouse, keyboard and colleagues (b) has wide field of view
	Wide field of view to increase immersion		
Cons	Difficult to interact with people or objects in the same room; some versions have complicated tracking set- up; may get VR motion sickness; may need connection to a computer with high powered GPU	Limited interaction options; limited field of view	Objects look slightly see- through in the display in (a) Has narrow field of view (52 degrees)
			On cheaper solutions of (b) pass through quality is poor (e.g., Quest)
			(b) can exhibit artefacts due to the real time processing

## Metaverse Environment

Metaverse environments offer potential advantages when compared to traditional 2D screen-based platforms for visualization in two key areas (Matthews 2018; Sommer et al., 2018; Hillmann 2021):

1) Enhanced remote sharing and collaborative opportunities offering benefits above traditional CAVE systems including affordability and interactivity (Cordeil et al., 2017).
2) Infinite space to visualize data where users show better recall (Krokos et al., 2019) and comprehension of scale (Lee et al., 2021).

There are several open-source (e.g., Vircadia) and commercial (Rec-Room, AltSpace, and Horizon Worlds) solutions. Many of these are based around entertainment (gaming, music, socialising) but increasingly work-based environments such as those provided by NVidia Omniverse, and Spatial.io will become more commonplace in the same way as Microsoft Teams and Zoom have for collaboration and communication. Metaverse environments offer excellent multidisciplinary collaborative possibilities. In the same way players interact in virtual gaming worlds (for example, as in Minecraft or Fortnite) collaborators from around the world can work in a shared, 3D virtual space using XR.

## Applications

There are an increasingly number of XR bioinformatics visualization applications which are reviewed in depth elsewhere (Goddard et al., 2018; El Beheiry et al., 2019; Calvelo et al., 2020; Venkatesan et al., 2021). Here we briefly overview XR tools but focus on the benefits of using these within the Metaverse.

### Imaging Examples

The 3D visualization capabilities of XR lend themselves well to image-based applications, where depth perception and ease of interaction with 3D objects can improve speed and accuracy (Timonen et al., 2021). As 3D tissue data analysis and reconstruction in spatial biology becomes feasible, this also opens up opportunities for XR visualization (Kuett et al., 2021). Applications such as ConfocalVR (Blanc et al., 2020b; Stefani et al., 2018), Syglass (Pidhorskyi et al., 2018), vLume (Spark et al., 2020), Genuage (Blanc et al., 2020a), DIVA (El Beheiry et al., 2020) allow loading of volumetric image data and point cloud data related to microscopy and 3D medical data.

### Non Imaging Examples

Single-cell technologies allow comprehensive transcriptional/epigenetic profiling of cell populations where a common step is to reduce the data to two or three dimensions (e.g., UMAP/tSNE) to generate “maps” of cells. 3D reductions can be useful for large and complex datasets to resolve overlapping clusters, and visualizing multiple reductions simultaneously can be very beneficial. CellexalVR (Legetth et al., 2021) is a VR application that does this while providing other tools to comprehensively visualize and analyze single-cell data. Others include Thiea (Bressan et al., 2021) which will also handle volumetric data, and singlecellVR (Stein et al., 2021) which offers visualization using Google Cardboard.

VRNetzer (Pirch et al., 2021) facilitates large biological and protein interaction network exploration, overcoming the often dense “hairballs” that are typical of such an analysis. ProteinVR (Cassidy et al., 2020) is a web-based tool that can visualise PDB protein structures on multiple devices using WebXR that gives useful biological context and allows users to situate themselves in 3D space. XR allows the layout of biological protein structures to be understood much more easily than a conventional 2D display (Wiebrands et al., 2018). iMD-VR (Deeks et al., 2020) shows VR is an effective and flexible method for interactive visualization of small molecule drugs docking into their protein targets. For chromatin visualization, CSynth (Todd et al., 2021) is a web-based tool which facilitates *de novo* modelling of complex 3D chromatin interactions in different disease tissues to understand how gene expression is affected by genomic structure.

### Combining Domains in the Metaverse

**FIGURE 1:**
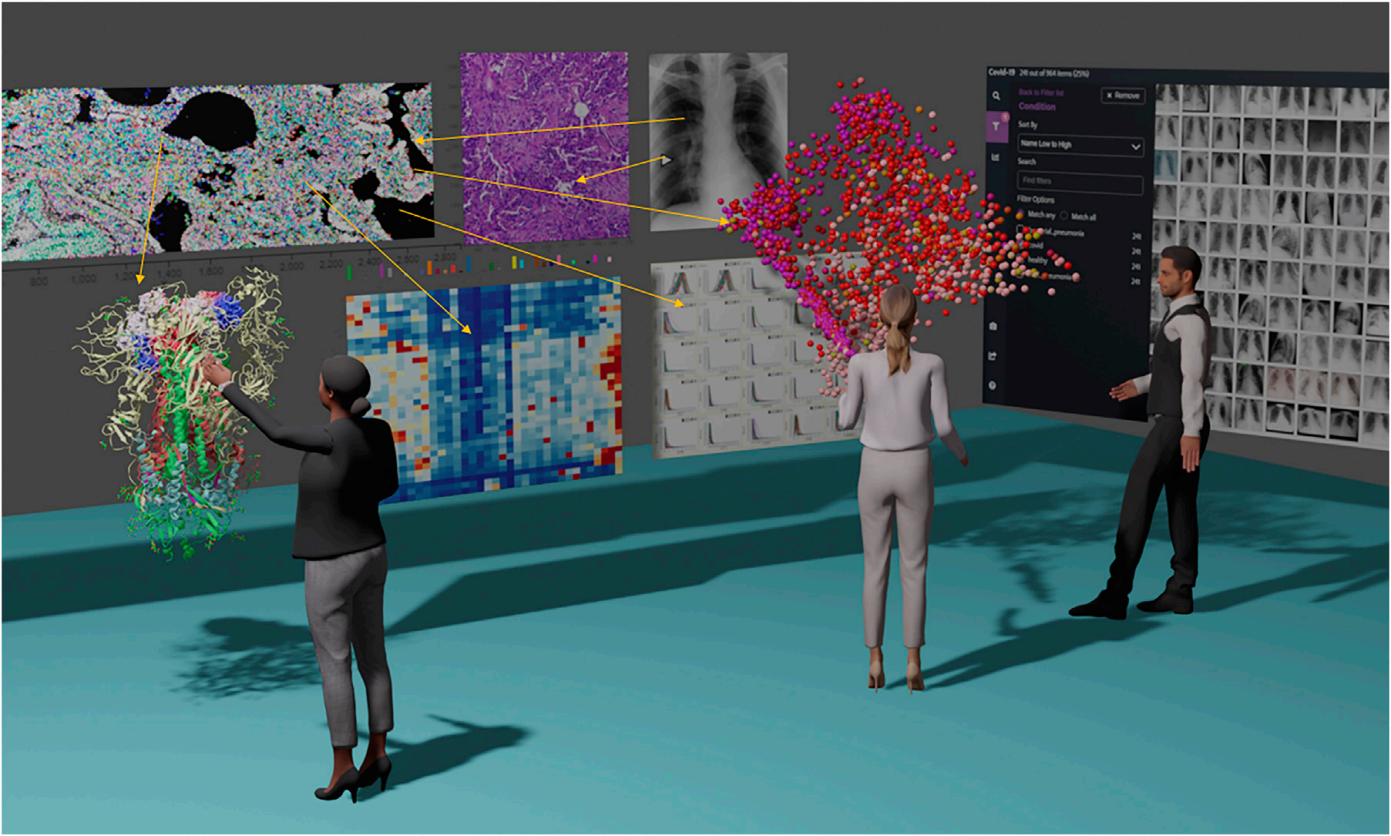
“Metaverse Evidence Wall” concept showing a combination of different packages and data types consolidated into a single space virtual environment. The example shows a multi-disciplinary group analyzing a fictitious COVID-19 spatial omics and single cell data set projected in 3D scatterplot. From left to right molecular imaging (with colored marker overlay), H and E stain, and patient cohort x-ray imaging are shown. Heatmaps derived from spatial analysis allow cellular phenotyping in conjunction with the location of the scatterplot and imaging. Meanwhile, a structural biologist manipulates the COVID spike protein in a XR protein viewer looking for variations in the patient’s genetic background that change binding affinities.

When working on multi-modal datasets the amount of dashboards, figures and information means screen real estate quickly runs out. This is less constrained in XR space and applications and could synergize if deployed together in the metaverse. The environment would allow clinicians, biologists, mathematicians and computational biologists from around the world to interact using an evidence wall or Anacapa chart based approach (Sparrow 1991). Anacapa charts are used in criminal investigations (and popularized in many crime TV shows) to communicate gathered evidence, timelines and relationships in a criminal case. This idea can be applied in the biological visualization to collate, organize and communicate information around a disease mechanism or biological phenomena. See Figure 1.

## Developing Metaverse Applications

There are three main choices: Unity, Unreal and Web-based frameworks. Unity and Unreal have a developer-friendly framework to allow multi platform XR apps to be developed and deployed reliably using the OpenXR standard (https://www.khronos.org/openxr/). This is currently at version 1.0 and is still evolving but greatly simplifies multi-platform development. Unity is generally regarded as the easiest development platform, although Unreal is more performant. Frameworks are required such as the Immersive Analytics Tool Kit (Cordeil et al., 2019) to help build high quality, interactive and scalable data visualizations in XR. For bioinformaticians there is a need to learn C# (Unity) and C++ (Unreal) in contrast to the more popular programming languages in data science bioinformatics such as Python and R which means developing such applications will be slower. One strategy is to deliver a client server approach where the data is generated using Python/R and then a Unity/Unreal client reads the data structures.

Many applications in bioinformatics visualization are developed for web browsers using JavaScript. Most devices already have a web browser and this approach offers a way to develop cross-browser applications. It also has the advantage that many bioinformatics visualization developers have experience of developing JavaScript applications and do not therefore have to learn a new language and development environment. In the past the reliability of these browsers with XR has been patchy, but this is stabilizing as it gains momentum. WebXR is an evolving standard that uses JavaScript and is available in an increasing number of web browsers including Firefox, Edge and Chrome. WebXR development’s main SDKs are AFrame and Babylonjs. These are built on a 3D javascript library called three.js which itself is built on WebXR. Aframe is very quick to learn but Babylonjs has an increasingly sophisticated toolset for 3D graphics such as node editors.

## Challenges in the Metaverse

### Connectivity and Infrastructure

Collaboratively working with colleagues around the world is a key feature of the metaverse concept, and will require communications infrastructure to be of a high standard. Latency in particular is as low as possible to preserve lifelike dialogue and social interactions which are hampered significantly when lag is present. This is not an issue just for developing nations, but any institute with ageing wifi/ethernet. A further challenge in a multi-vendor, Metaverse environment is a single sign-on experience that will allow a seamless connection between multiple users. Vendors such as Microsoft may have the advantage here since the Hololens 2 uses a Windows OS variant allowing single sign-on across devices within organizations.

### Future Legislation

Early problems have been widely reported in the media where under-aged individuals have been using Meta’s platform, plus, there have been many reports of verbal and “physical” abuse of individuals. To counter this, Meta has introduced a minimum 2 m rule between participants, but the question remains as to whether legislation will be introduced to enforce this (and other rules), and if so, will these laws also need to be enforced in STEM-centric metaverses?

### Hardware and Design

A well designed VR environment is essential for a comfortable user experience as data analysis and visualization can be more intense. Some users can be in VR for hours at a time, some will only manage a few minutes without sickness/fatigue, but this is often indicative of a poorly constructed and lit environment. Key is the frame rate which should be at least 72 FPS (ideally between 90 and 120 FPS) to ensure a smooth experience. Also, allowing the hardware to be inclusive for all people including disabled groups will also need to be addressed but offers huge potential so everyone in the metaverse has equal access. Towards that, HMDs are gradually becoming smaller and lighter, incorporating features such as eye and facial expression tracking. It should also be ensured that all environments are designed so individuals can use it in a sitting position.

The preferred solution is a low-cost untethered experience but the downside of all untethered XR devices is they have limited CPU/GPU power which is a major issue for visualization as data sets become larger. For example, it is not uncommon in a single cell experiment to have hundreds of thousands of data points, all of which may have many columns of metadata. Displaying such data on a portable and relatively under-powered device is very challenging.

A potential solution is to stream applications on demand. 3D “remote desktops” will allow large datasets to be rendered in the cloud and streamed on low powered devices. Cloud XR (https://developer.nvidia.com/nvidia-cloudxr-sdk) leverages cloud-based GPUs to render images from the application and uses fast, low latency networks (such as Wifi6/5G) to stream the data to any HMD with a client. With a good network a user can interact with the application as if they are running on a local machine. This approach is challenging because unless the latency is kept below 20 ms the user may experience immersive sickness. Microsoft has a technology called Azure Remote Rendering (ARR) which works in the Hololens 2 but is bespoke for particular objects.

### Software Design

The different software environments that each particular HMD provides scope for innovation, but with this flexibility comes a danger that users may need to learn how different systems work for each application. Coupling that with a 3D environment means the user experience could be complex and confusing for first time users. For example, bioinformatics browser-based Javascript front-ends are often developed using React which allows certain functionality to be quickly developed with similar style, MacOS has “Themes”, Windows has “Microsoft Style Guides”. The 3D space around the user may become cluttered with dashboards. More research is required into machine learning based automated layout methods that extract features from the dashboards combined with human-in-the-loop processes to help group, hide and remove non-essential items. With no definitive single company in the 3D space this guidance is lacking although there are emerging standards such as IEEE SAA.

Applications may need to provide interactive tutorials where users are walked-through the features to address this. There needs to be much clearer guidelines and style guides as to the best way of constructing such interfaces. Also the fact the number of HMDs is small compared to traditional computers means that user testing can be more limited.

As new XR systems are released into the market, developers will have a responsibility to ensure their software is compatible with these units. This mostly applies to the hand controllers and which software, or runtime, is used (SteamVR, Windows Mixed Reality, Oculus link). New virtual models may also need to be made for aesthetic purposes, and if needed, functionality mapped to the new button/touchpad layouts. OpenXR aims to solve these issues, but as mentioned earlier is still in its infancy.

### Publication of XR Tools

Most academic researchers are judged on the output of peer-reviewed publications to secure further funding. Journals often require the source-code (or at least the application) to be available so it can be installed and tested by reviewers, but with XR applications there is an obvious complication in that not all reviewers will have access to HMDs which could hinder their ability to effectively assess the work. To facilitate review, authors will often provide detailed supplementary videos to show how their software performs, but this still requires reviewers to use a little imagination. Until XR is more widespread, journals need facilitate the review by sending XR equipment to authors and the reviewers *via* their editorial office to maintain reviewer anonymity. As the development of XR applications continues it may be necessary for journals/publishers to have defined guidelines saying how they will deal with XR submissions, but in the meantime it is up to the authors to determine if their journal of choice will do this before submission. In the experience of the authors of this article, some people are naturally skeptical about XR and what more it can offer, but often have a far more positive view after testing it.

### Evaluation of XR Tools

Another issue is benchmarking. XR has potential advantages over traditional approaches for speed, reproducibility and accuracy but comparing these in a systematic unbiased way requires careful consideration. A good example of a quantitative way of measuring effectiveness of an XR application has been shown using AR for head and neck carcinoma (Gsaxner et al., 2021) where 11 experts were surveyed and training and testing times were compared between XR-experienced users and non-experienced users. This study also used the System Usability Scale (SUS) (Sauro and Lewis 2016).

### Non Spatial Data

Non spatial data, such as descriptive text, tables, 2D images and multimedia sources, will need to be accessed in the metaverse. MR and AR systems allow the outside world to be viewed and potentially augmented. For example, highlighting a 2D word such as “SARS-CoV-2 variant” in AR or MR could pop up a 3D overlay on the text, showing the image of the virus and structural variations. An example of using an AR framework to show associated imagery is Schol-AR.

When immerse in VR, reading comprehension of 2D screens has been shown to be non-significant but response times to answering multi choice questions has been shown to be 10% slower (Rau et al., 2018). New methods to ingest data need to be developed to improve comprehension. For example, Rapid Serial Visual Presentation (RSVP) was tested in VR where text was displayed word-by-word briefly at a fixed location. When moving in virtual space this proved more effective for comprehension since the user could focus in one area (Rzayev et al., 2021. Conversely, a large wall of related images (as in Figure 1) could be viewed in VR without the constraints of a small 2d screen, allowing more data to be viewed at once.

Given its importance, more research needs to be done to understand how to comprehend and access non spatial data sets and the cognitive load in different use cases.

### New Ways to Interact With Data

Speech to text is the obvious way to enter data as opposed to typing, but could also be used to activate features rather than using a menu in the VR environment. Wrist-based “Brain Computer Interfaces” or BCI such as Neuralink (Musk and Neuralink 2019) offer a tantalising glimpse that data querying and selection could be achieved by thought power. Electromyography (EMG) uses wrist-based bands that can sense motor neuron signals, turning them into gestures and controls.

### Haptic Devices

Haptics gloves are at an early stage of development (Fang et al., 2020; Preechayasomboon and Rombokas 2021) and (https://github.com/LucidVR/lucidgloves) would allow objects in the metaverse to be physically interacted with. For example, a cluster in a 3D scatter plot could be physically grabbed and isolated for further analysis. Mixed reality systems allow surfaces such as walls and table tops to provide tactile feedback. Such interactions make a more compelling immersive experience and make selection more accurate, in the same way that drawing in the air is not as accurate as drawing on a physical surface.

## Discussion

The metaverse has been heralded as the next platform in computing and the future of the internet. In the 1980s, few predicted the revolution of most people having their own personal computer. 40 years on computers and mobile devices are well established platforms. A key factor is development of XR hardware that each have different features and tradeoffs (such as display, weight, price etc). Can XR wearables become inexpensive and light enough so that they become part of an everyday experience whilst respecting privacy (Lebeck et al., 2018; Ridel et al., 2016)? Visualization research offers exciting possibilities for maximizing the metaverse’s promise. The burgeoning need to analyze complex multimodal biological datasets with multidisciplinary teams from different institutes has become the norm and the metaverse should surely leverage that.

There will need to be a transition from the 2D desktop into more immersive environments. MR may win here over VR since MR allows better interaction with the surrounding environment. The fact the user can see a mouse and keyboard potentially allows more accurate control and data entry compared to VR, and may give MR the edge in bioinformatics visualization. New devices are being developed that can switch between MR and VR which offer the best of both worlds (e.g., Varjo, *Lynx* R1).

As data sets increase in size, experiences will need to be generated from very powerful local or, more likely, cloud-based systems. This will likely need better infrastructure but will allow data visualization to be constrained only by the data scientist’s imagination and promises new ways of interacting with and communicating data. More work needs to be done exploring the use case transitioning 2D to 3D workflows, so 3D interaction is used appropriately. The advantage of remote collaborations, 3D and hand/gesture-based interactions, combined with virtual, huge screen real estate, will open up new ways of working in a variety of bioinformatics visualization scenarios. With access to large multi-dimensional visualizations, more work will need to be done to assess cognitive load and human computer interaction to ensure humans are at the center of the systems we build. It will require us to think beyond the current 2D paradigms which have dominated the computer industry and embrace the advantages that the metaverse may bring.

## Data Availability

The original contributions presented in the study are included in the article/Supplementary Material, further inquiries can be directed to the corresponding author.
